# De Novo Synovial Chondromatosis following Primary Total Knee Arthroplasty: A Case Report

**DOI:** 10.3390/life13061366

**Published:** 2023-06-10

**Authors:** Sebastian Braun, Dimitrios A. Flevas, Ruba Sokrab, Robert G. Ricotti, Carolena Rojas Marcos, Andrew D. Pearle, Peter K. Sculco

**Affiliations:** 1 Department of Orthopedics (Friedrichsheim), University Hospital Frankfurt, Goethe University, 60323 Frankfurt am Main, Germany; 2 Stavros Niarchos Complex Joint Reconstruction Center, Hospital for Special Surgery, New York, NY 10021, USA; flevasd@hss.edu (D.A.F.); sokrabr@hss.edu (R.S.); rojasmarcosc@hss.edu (C.R.M.);; 3 Department of Orthopaedic Surgery and Sports Medicine, Hospital for Special Surgery, New York, NY 10021, USA; pearlea@hss.edu

**Keywords:** synovial chondromatosis, total knee arthroplasty, knee osteoarthritis, knee joint, benign soft tissue tumor

## Abstract

In this case report, we present a rare case of a female patient who developed pain and swelling after a total knee arthroplasty. An extensive diagnostic workup including serum and synovial testing to rule out infection was performed in addition to advanced imaging including an MRI of the knee, but it was only after an arthroscopic synovectomy that the diagnosis of secondary synovial chondromatosis was confirmed. The purpose of this case report is to highlight the occurrence of secondary synovial chondromatosis as a rare cause of pain and swelling after total knee arthroplasty, thereby assisting clinicians in providing prompt diagnosis, surgical treatment, and efficient recovery in the setting of secondary synovial chondromatosis after total knee arthroplasty.

## 1. Introduction

Synovial chondromatosis (SC) is a rare, proliferative but benign disease of the synovial membrane of joints, tendon sheaths, or bursae that can cause severe dysfunction of the affected synovial joints [1]. SC is most common in men, typically presents during the third to fifth decade of their life, and primarily affects large joints such as the knee or hip [2,3]. The synovial membrane is transformed into multiple cartilaginous nodules due to metaplasia [4]. Often, these cartilaginous nodules detach and form intra-articular loose bodies, while the synovial fluid nourishes their growth [5]. The formation of multiple chondroid nodules, osteochondral, or osseous loose bodies is the result of the disease process and usually presents as a monoarthropathy [1,6,7]. When it involves the knee joint, SC is usually restricted to the anterior compartment of the knee and includes the anterior fat pad and suprapatellar pouch [8]. Regarding symptoms, SC in the knee joint is characterized by non-specific symptoms that include pain, swelling, restricted range of motion, locking, giving away, and crepitus [9].

The disease presents in the following two forms: primary SC and secondary SC. The etiology of the primary SC is not well understood. Secondary SC is thought to occur as a result of mechanical changes in a joint due to an arthropathy such as osteochondrosis dissecans, recurrent trauma, or previous surgical procedures [1,7]. The diagnosis of SC, in either form, is often missed or delayed due to the fact that symptoms are non-specific and insidious in the onset [5]. While clinical and radiological findings are useful indicators of secondary SC, a histopathological analysis is required for diagnostic confirmation.

In this paper, we report a rare case of a female patient who developed pain and swelling after a total knee arthroplasty (TKA). An extensive diagnostic workup including serum and synovial testing to rule out infection was performed in addition to advanced imaging including an MRI of the knee, but it was only after an arthroscopic synovectomy that the diagnosis of secondary SC was confirmed. Despite the wealth of research on SC, our paper highlights a rare instance of secondary SC emerging after TKA, a situation infrequently noted in the scientific literature. The distinctiveness of our case presentation resides in the examination of this unusual clinical presentation, compounded by the patient’s pre-existing rheumatoid arthritis (RA). Our aim is to guide medical professionals toward a swift diagnosis and the effective management of secondary SC following TKA.

## 2. Case Report

**Figure 1:**
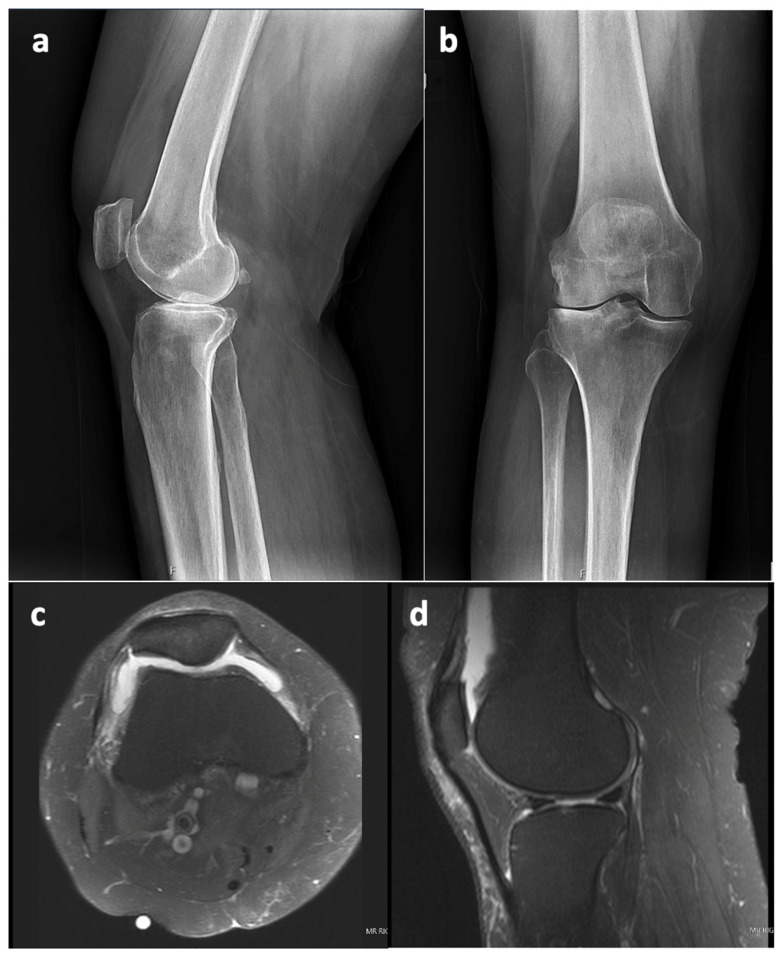
X-ray from 11 June 2018 ((**a**)—lateral view, (**b**)—anterior–posterior view) and MRI from 13 March 2017 ((**c**)—axial view, (**d**)—sagittal view); right knee shows knee osteoarthritis with no sign of synovial chondromatosis prior to TKA in August 2018.

This case report refers to a 62-year-old woman who received a right TKA for primary osteoarthritis (OA) in August 2018 at the age of 58 years at an outside hospital. Informed consent was obtained from the patient with an explanation that the data concerning her case would be submitted for publication. The patient’s x-ray and MRI images showed no evidence of SC prior to her TKA surgery, as seen in Figure 1. The synovial tissue samples of the knee joint obtained during her index right TKA surgery also showed no evidence of SC, but showed synovial hyperplasia with lymphocytic infiltration, as this can occur in the context of degenerative joint disease or in association with RA.

Her early recovery after primary TKA was complication-free. However, four years later (April 2022), the patient presented with an insidious onset of swelling, effusion, and pain in her right knee. The pain was described as an 8/10 on the numeric rating scale, non-radiating, worsened with certain movements, and improved with rest. The patient tried rest and pain medication, which provided minimal relief. She underwent an initial evaluation to determine the cause of her knee problems, particularly, the possibility of periprosthetic joint infection (PJI). The patient had a history of rheumatic disease and antiphospholipid syndrome, and other comorbidities are shown in Table 1.

**Figure 2:**
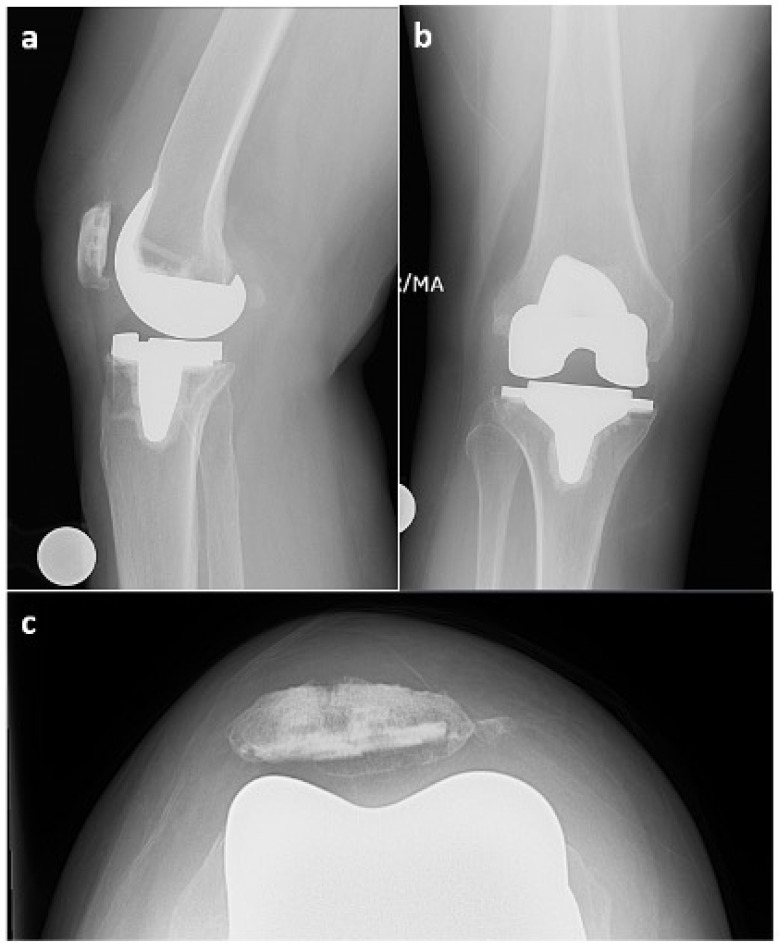
X-rays from 9 September 2022 ((**a**)—lateral view, (**b**)—anterior–posterior view, (**c**)—Merchant view). Right total knee arthroplasty (TKA) was reidentified with components in anatomic alignment without fracture.

There were no systemic symptoms indicative of infection such as a history of fever or chills. An examination revealed an antalgic gait, diffuse moderate knee swelling with associated warmth, a range of motion of 0 to 100° flexion without an extension lag, no ligamentous instability, and a neutral limb alignment. Her laboratory values were as follows: erythrocyte sedimentation rate (ESR) 124 mm/h (reference range 0–20 mm/h), C-reactive protein (CRP) 17.7 mg/dL (reference range: 0.0–1.0 mg/dL). A knee aspiration was performed, which demonstrated 25 mL of straw-colored native fluid, 77% neutrophils, and negative cultures after 14 days. NexGen sequencing (MicroGenDx, Orange Country, FL, USA) was also performed on a second aspiration, and the aspiration was also negative for bacterial DNA fragments. The radiographs were normal and showed no evidence of implant loosening (Figure 2). Several aspirations were performed previously that showed no evidence or signs of PJI.

**Figure 3:**
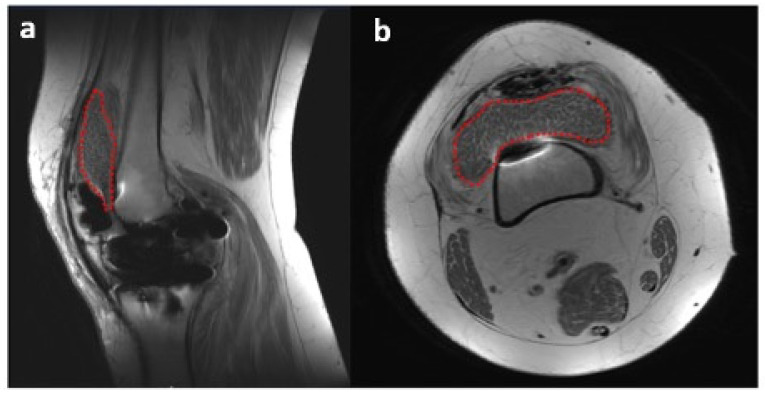
MRI of right knee 3 October 2022 (GE, Coil: HD TRknee PA): (**a**)—sagittal plane (proton density—PD), (**b**)—axial plane (PD). Dotted red line outlines the appearance of diffuse rice bodies in the anterior compartment and medial and lateral gutters.

An MRI of the right knee was also performed and demonstrated pronounced debris-containing synovitis with the appearance of diffuse rice bodies in the anterior compartment and medial and lateral gutters (Figure 3). Distinguishing SC from rice bodies in the context of a PJI can be challenging. Rice bodies and SC both involve the formation of loose bodies, yet their origin and associated conditions vary. Additionally, the MRI appearances can be similar, making it hard to distinguish between the two. Both conditions can appear as multiple bodies within the joint space, with signal characteristics that can overlap. On the MRI, PJIs present as effusion and potentially synovial thickening, which might also complicate the distinction between rice bodies and synovial chondromatosis. In such complex scenarios, a histopathological examination is often necessary to confirm the diagnosis. The radiology report in this case stated that the presence of rice bodies (rice bodies are described as iso- and hypointense to skeletal muscle tissue, whereas SC would appear slightly hyperintense [10]) was compatible with the clinical concern of infection. However, the ability to rule out PJI was complicated by the patient’s elevated ESR and CRP and the diagnosis of anti-phospholipid syndrome with positive rhetological markers (ANA+).

Due to persistent knee pain, ongoing synovitis, and the need for a tissue biopsy, the patient was indicated for arthroscopy, synovectomy removal of multiple loose intra-articular rice bodies, and biopsy to further assess the possibility of chronic PJI and overall decompression.

**Figure 4:**
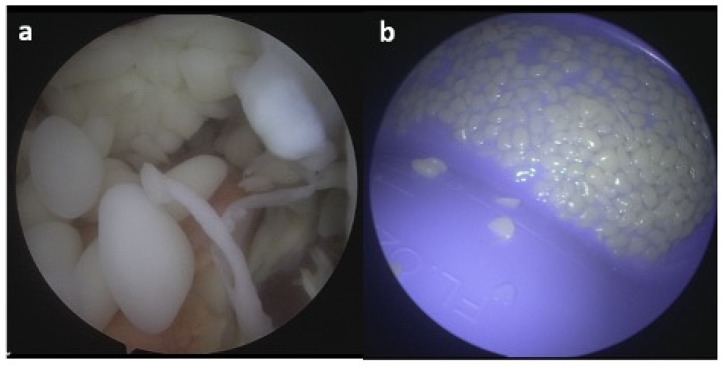
Arthroscopic images 24 January 2023; (**a**)—intra-articular view of loose rice bodies and signs of synovitis, (**b**)—rice bodies after arthroscopic extraction.

**Figure 5:**
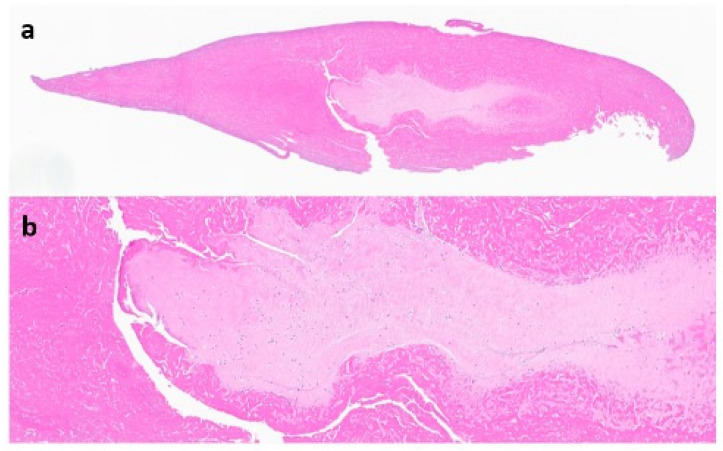
Microscopic display of a loose body after paraffin-embedded sectioning (**a**), hematoxylin and eosin (HE) stain, 20× magnification (**b**).

The suprapatellar pouch was found to be filled with a large number of smooth, white and loose rice bodies. The synovial fluid and rice bodies were collected for pathology and culture (Figure 4). The remaining loose bodies were excised from the knee using a grasper, arthroscopic shaver, and bipolar electrocautery. In addition to rice bodies, there were extensive synovitis and adhesions present, which were excised with a combination of an arthroscopic shaver and bipolar electrocautery. Given the patient’s need for anticoagulation due to a history of DVT and APS, care was taken to cauterize all possible bleeding sources within the knee. The implant interfaces were carefully examined, and they were all found to be well fixed. An arthroscopic synovectomy was performed, leaving the implants in place. A pathological examination revealed chondroid metaplasia covered by fibrin-like material (Figure 5). Upon careful histopathological examination by our pathologist, the structures that were originally identified as rice bodies were in fact small loose bodies from SC. This unusual case represents a rare instance where SC was initially mistaken for the more commonly seen rice bodies found in RA.

After an uneventful postoperative hospital stay, the patient was discharged. The results of all the intraoperative cultures were negative. The patient’s subsequent hospital course and recovery were characterized by mild pain and swelling, which improved significantly as compared to her pre-operative condition. The mobility of the knee was 3–120 degrees of flexion. There was no ligamentous instability, as well as no tenderness to palpation. A five-month follow-up revealed stable clinical progress without recurrence, affirming the effectiveness of the surgical intervention. In addition, the clinical scores notably improved from the pre-operative assessments to this final follow-up: the pain level (numeric rating scale) dropped from a pre-op value of 8 to 0, and the PROMIS-10 global health physical score, a measure of the patient’s perceived physical health, also showed substantial improvement, rising from a pre-op value of 26.7 to 44.9.

## 3. Discussion

Although little is known about the etiology of primary SC, the research suggests its association with chromosomal alterations [11]. The underlying pathophysiology is believed to be a metaplastic transformation of the synovium to the foci of hyaline cartilage tissue. The conversion to malignancy was reported but is considered rare with an estimated risk of 5% [12,13]. Although arthropathy is causative in the secondary form, its subsequent occurrence after the implantation of a TKA is not well understood and is extremely rare. This condition is likely to emerge in response to pre-existing joint abnormalities or alterations, such as those induced by surgical procedures such as TKA. The mechanical shifts within the joint caused by these procedures might instigate synovial metaplasia, culminating in the formation of cartilaginous nodules. As such, SC post-TKA signifies a secondary manifestation of this rare pathology, which is typically observed in an older demographic compared to primary SC [1]. In 1977, Milgram [3] divided SC into the following three succeeding stages: (1) intrasynovial disease without loose bodies, (2) transitional disease with active intrasynovial proliferation and free loose bodies, and (3) multiple free loose bodies without active intrasynovial proliferation. SC clinically manifests with synovial thickening, crepitations, palpable loose bodies, and a limited range of motion. Because symptoms are relatively non-specific and have an insidious onset, diagnosis is often delayed and takes up to five years on average [14]. These loose bodies may be present upon radiographic examination and appear as multifocal, rounded, articular, or periarticular calcifications of varying sizes in a joint [15]. Because calcification and mineralization are delayed in up to 20% of cases, we suggest that the diagnosis should not be based on plain radiographs alone. An MRI of the affected joint allows for a better assessment of the cartilaginous process and the extent of involvement in the joint and surrounding tissues. Synovial chondromatosis is usually monoarticular, although multiple joints are affected in rare cases [1]. Men are up to four times more likely to be affected by synovial chondromatosis than women. The most commonly affected joint is the knee, with approximately 70% of all manifestations [13]. While the typical age of manifestation ranges from 30–60 years [2,3], the secondary form is more common in older individuals and usually occurs in the sixth to seventh decade of life [1].

The treatment goals of SC are to prevent recurrence and to delay the progression of secondary joint damage. There is no clear-cut decision on the therapy for SC. The need for complete or partial synovectomy is controversial [15,16], although it appears to be the primary treatment indication for pathology originating from the synovium. In the past, several studies supported the isolated removal of loose bodies without additional synovectomy, as no superiority exists for such an adjunct treatment [4,17]. Shpitzer et al. [17] showed that the recurrence rate with and without synovectomy is not significantly different. Other studies supported that synovectomy is important in preventing the recurrence of SC [18,19,20]. In addition, there is controversy as to whether synovectomy should be performed through an open approach or arthroscopically. Open synovectomy provides a better visualization of the articular surfaces; alternatively, arthroscopic techniques result in lower morbidity, faster rehabilitation, shorter hospital length of stay, and less postoperative pain [21,22].

As in our case presentation, the first-line treatment for secondary SC should be the use of anti-inflammatory drugs [1]. In SC that does not respond to conservative treatment, TKA is usually recommended as the treatment of choice. Because our patient developed SC following TKA, the best treatment option was more challenging. A study performed by Ackerman et al. [23] investigated the outcomes of seven patients undergoing primary total hip arthroplasty (THA) and four patients undergoing primary TKA for end-stage OA associated with SC. They were able to show that the recurrence of SC after total joint replacement is relatively frequent with 25% (one patient) after TKA and 14% (one patient) after THA. In this small cohort of patients, the transferability of the results for patients post-TKA is difficult. Still, the results of the literature remain valuable due to the limited data of this rare condition. A larger retrospective study of 20 patients who received TKA for SC were reviewed over an average follow-up period of 7 years by Houdek et al. [24]. They were able to demonstrate a similar disease-free survival of 78%. A study by Chong et al. [25] described a 5-year follow-up after radiotherapy for chronic recurrent SC of the knee. The authors compared the efficacy of this radiotherapy with that of heterotopic ossification (HO), as SC and HO are both characterized by increased osteoblastic activity. The patient in this study underwent multiple surgeries prior to radiotherapy to the knee within a few years. Radiotherapy immediately followed the last surgery. During the follow-up period of 5 years after radiotherapy, there was no recurrence or progression of SC. The most radical treatment option for end-stage OA or chronic recurrent SC is arthrodesis of the affected joint. However, Church et al. [26] also demonstrated that despite arthrodesis, a patient with highly refractory SC may experience a recurrence of SC.

SC after TKA may also appear like chronic PJI as it presents with pain, swelling, and stiffness. Infection should be ruled out with a serum marker analysis and synovial fluid aspiration. In this case, the patient’s history of APS and elevated ESR and CRP made ruling out PJI even more challenging. Currently, the MSIS criteria to diagnosis PJI after TKA is not as accurate when applied to patients with inflammatory conditions [27,28]. Subsequently, NexGen sequencing was used to further rule out the presence of PJI, and the results were negative. Future research is needed to better understand the value of NexGen sequencing to rule out PJI in patients with inflammatory arthritis and elevated serum markers. The patient’s medical history and symptoms warranted an evaluation by a specialist in rheumatology. Upon a comprehensive review and examination, a diagnosis of RA was confirmed, which is notable given the recognized association between SC and discoid lupus erythematosus (DLE). However, in this particular case, there were no clinical indications of DLE. While DLE could have potentially provided an alternative explanation for the observed SC, without the presence of relevant clinical indicators, a diagnosis of DLE could not be substantiated. This patient’s situation is intriguing, especially considering the case report by Suyama et al. [29], which described an association between SC and lupus erythematosus. However, based on her medical history, current clinical signs, and blood work, she does not meet the diagnostic criteria for lupus. The patient’s blood work reveals a positive ANA, which is commonly observed in many rheumatological diseases including lupus. Despite this, her negative complement C3 and C4 levels, rheumatoid factor, Sjogren’s antibodies, CCP antibodies, double-strand DNA, Smith, and Smith/RNP antibodies all suggest that lupus is unlikely. Therefore, we attribute her positive ANA to be more likely due to her antiphospholipid syndrome than lupus. Nevertheless, the fact that the patient’s mother was diagnosed with lupus is noteworthy. In addition, the appearance of rice bodies on MRI was also associated with infectious etiology such as tuberculosis [30]. In this particular case, these loose bodies were determined to actually represent nodules of SC, diverging from the typical rice bodies associated with conditions such as RA. Despite both SC and rice bodies inducing similar clinical symptoms, their distinct histological attributes necessitate meticulous pathological examination for accurate diagnosis. This distinction, which is critical in formulating the appropriate management approach, underscores the need to consider surgical interventions such as synovectomy or loose body removal, instead of solely addressing inflammation through medical treatment. As these rice bodies were in fact small SC loose bodies, we hope this case report will heighten the awareness of this potential correlation, encouraging clinicians to consider SC as an alternative diagnosis when confronted with similar scenarios.

Our findings indicate that the patient exhibits signs of secondary synovial chondromatosis, which occurred after the TKA surgery. To the best of our knowledge, there is no evidence in the literature to suggest that this was reported previously. Therefore, our paper provides valuable insights and contributes to the literature by reporting this rare occurrence.

## 4. Conclusions

In summary, SC following TKA is an exceedingly rare condition. The possibility of SC should be considered in patients who were ruled out for infection and present with chronic, painful swelling and effusion of a knee following TKA. Furthermore, the current literature recommends various and controversial treatment options for SC in a native knee with regard to the recurrence of SC. Due to the rarity of this case, it is difficult to determine an optimal treatment strategy for primary onset SC after TKA. This case underscores the importance of considering secondary SC in the differential diagnosis of patients presenting with persistent joint symptoms post-TKA. Our findings also highlight the potential of arthroscopic synovectomy in the definitive diagnosis and management of such cases. The uniqueness of this topic lies in its rarity and the diagnostic challenge it presents, necessitating further research to better understand its pathophysiology and optimize its management.

## Figures and Tables

**Table 1:** Past Medical History/Comorbidities.

Past Medical History
Rheumatoid arthritis (RA) negative for rheumatic factor Antiphospholipid syndrome (APS) Asthma Clotting disorder Deep vein thrombosis (DVT) Gastroesophageal reflux disease (GERD) Transient ischemic attack (TIA)/stroke (2015) Total knee arthroplasty (TKA) 08/2018

## Data Availability

The data presented in this study are available upon request from the corresponding authors.
